# Prompt Reform Needed in Poor-Law Nursing of the Sick

**Published:** 1913-06-14

**Authors:** 


### PROMPT REFORM NEEDED IN POOR-LAW NURSING OF THE SICK.

The inaction of the Government in relation to the costly Royal Commission's Report on the Poor Law, whatever its cause, cannot justify any longer delay in the enforcement of the reforms urgently demanded in the present Poor-Law nursing arrangements. We called attention in The Hospital of April 5, 1913, to the important points raised by the Draft Order, and strongly commented upon its inadequacy and the ignorance it exhibited of the present requirements and position of sick nursing in well-managed institutions. For upwards of thirty years the Workhouse Nursing Association has laboured assiduously to improve the nursing of the sick in Poor-Law institutions. It has supplied upwards of 880 well-trained nurses and been the means of raising many thousands of pounds in furtherance of these objects. These nurses have done quietly useful work in our rural Unions especially, and we are glad to learn that the Association recently submitted an important memorandum to the Poor-Law Orders Committee at the Local Government Board, suggesting amendments to several of the provisions of the Poor-Law Institutions Draft Order referred to, which this Committee has in hand. The Draft Order, as it stands, would prove retrograde rather than progressive, so far as the nursing administration in rural workhouse infirmaries and sick wards is concerned.

A further deputation from the Workhouse Nursing Association, consisting of Mrs. John Talbot; Miss Gibson, late matron of the Birmingham Infirmary; Mr. H. D. Kimber; and the Secretary, had a lengthy interview with the Poor-Law Orders Committee and emphasised and explained fully the views of the Workhouse Nursing Association, and the seriousness of their objection to the Draft Order as it stands. They also urged the importance of giving immediate effect to a number of alterations in the proposed Order which they have formulated in their carefully drawn memorandum.

This matter is one which demands the immediate personal attention of Mr. John Burns as President of the Local Government Board. The position is serious from the public's point of view and is one that must excite much attention in the press, unless it is dealt with adequately, and quickly. We are confident that Mr. Burns if he would devote half an hour to the subject, could put everything right and see that an adequate Order was issued immediately. The only axe the Workhouse Nursing Association has to grind is the better care of the sick poor, who are now more than formerly forced to enter Poor-Law institutions, which at present are under-staffed and where the nurses are so overworked as to be falling ill in consequence. Wandsworth at the present time, we understand, is a sad example of such under-staffing and overworking. Miss Wamsley has done good service in her two recent reports. That on the Newmarket Union Workhouse exhibits in detail how bad the nursing arrangements are at present. Mr. Burns, we have no doubt, realises as much as we do that the public are smarting, because, despite the heavy rates they now have to pay, there is an increasing volume of evidence that the sick poor, owing to inadequate nursing, are at present sadly neglected in many Poor-Law institutions, for the efficiency of which Parliament holds Mr. John Burns directly responsible. We beg of him, therefore, to give his personal attention at once to this matter, and to see that it is set right and that the sick nursing in Poor-Law institutions is put upon a proper and adequate basis without a moment's avoidable delay.

